# Chronic recurrent multifocal osteomyelitis of clavicle: a rare isolated location (a case report)

**DOI:** 10.11604/pamj.2023.46.53.39452

**Published:** 2023-10-12

**Authors:** Khaled Kamoun, Wajdi Arfa, Malek Ben Chaalia, Wajih Oueslati, Leila Abid, Mourad Jenzri

**Affiliations:** 1 Pediatric Orthopedic Department, Kassab Institute, El Manar University, Tunis, Tunisia,; 2 Anatomopathology Department, Kassab Institute, El Manar University, Tunis, Tunisia

**Keywords:** Osteomyelitis, chronic, inflammatory, clavicle, case report

## Abstract

Chronic recurrent multifocal osteomyelitis (CRMO) is a rare disease. It is a non-microbial inflammatory bone affection that occurs more often in children with insidious onset and non specific presentation making diagnosis challenging. This study reports a case of CRMO with an unusual location. A 9-year-old child had a painful swelling over the medial side of clavicle with fixed mass. Radiographs showed osteolytic lesion on the medial part of clavicle extending to the acromioclavicular joint with soft tissue edema in magnetic resonance imaging (MRI). No inflammatory markers in biological exam. Needle biopsy, initially performed, suspected bone infection but children didn´t recover after 2 weeks of antibiotics. Surgical biopsy, histology sections were compatible with CRMO diagnosis. Children received a non steroid inflammatory drug with positive response, pain relief and decreasing of the clavicle swelling. CRMO should be suspected and biopsy is some time helpful in such unusual location.

## Introduction

Chronic recurrent multifocal osteomyelitis (CRMO) is a rare non-microbial inflammatory bone affection [1]. It occurs preferentially in children and young adults [2]. Autoimmune is the most suggested etiopathogenesis [3]. It usually manifests by multifocal bone pain with insidious onset and recurrent evolution. Osteolytic lesions are usually observed in affected bones on radiographs. It often constitutes an exclusion diagnosis after eliminating malignant tumors and bone infections. This case aimed to highlight diagnosis difficulties in such a rare location in which bone biopsy was contributive.

## Patient and observation

**Patient information:** a 9-year-old girl with no previous medical history was seen in the outpatient department for a painful swelling over the left clavicle that had been evolving over a month with no history of trauma.

**Figure 1:**
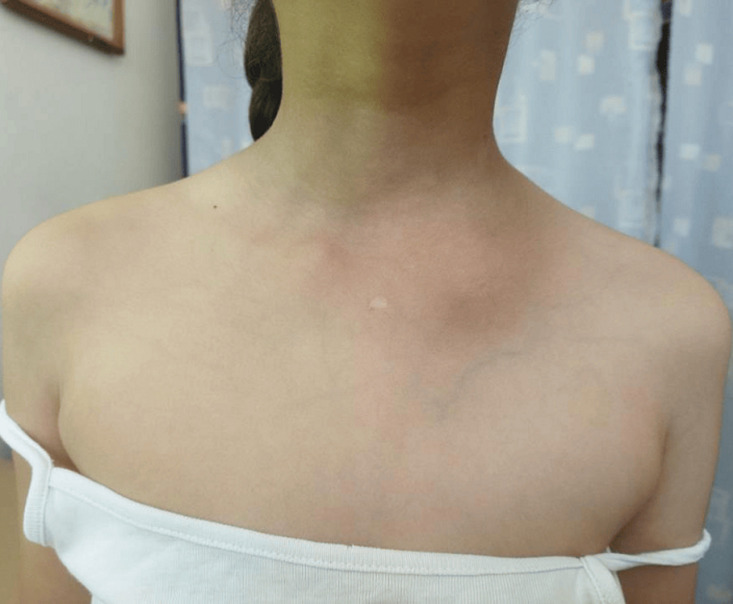
clinical image of shoulders: swelling above the left clavicle with skin redness

**Clinical findings:** physical exams found a girl with good general condition and with no fever. A swelling above the proximal quarter of the clavicle was noted, which was firm, painful, and fixed to the deep plane with skin redness (Figure 1). Shoulder motion was limited by pain.

**Timeline of current episode:** in April 2022 child presented swelling and pain over her left clavicle. X-ray, ultrasound, and MRI were performed in addition of biological markers. First needle biopsy then surgical biopsy in May 2022 with histology section, immunohistochemical study. The patient was referred to the rheumatology department. Bone scintigraphy searching for other locations. Non-steroidal anti-inflammatory drug (NSAID) treatment, July 2022 decreasing of clavicle swelling and pain relief.

**Figure 2:**
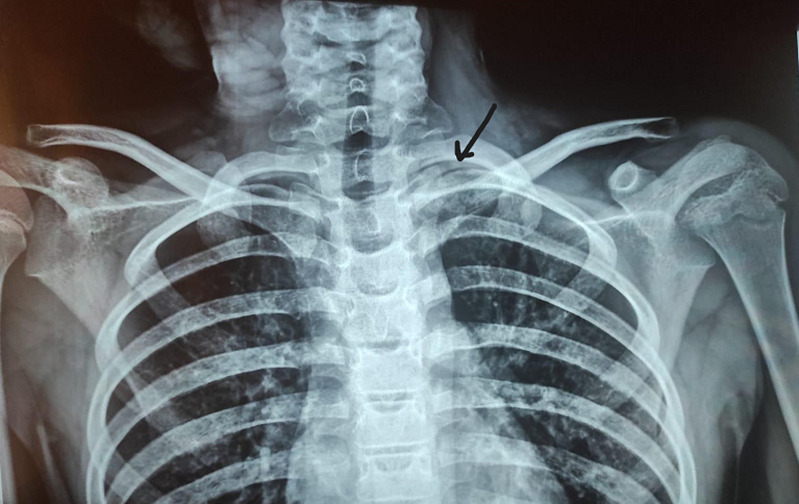
anteroposterior shoulders X-ray: osteolytic bone lesion with cortical disruption involving proximal part of left clavicle

**Figure 3:**
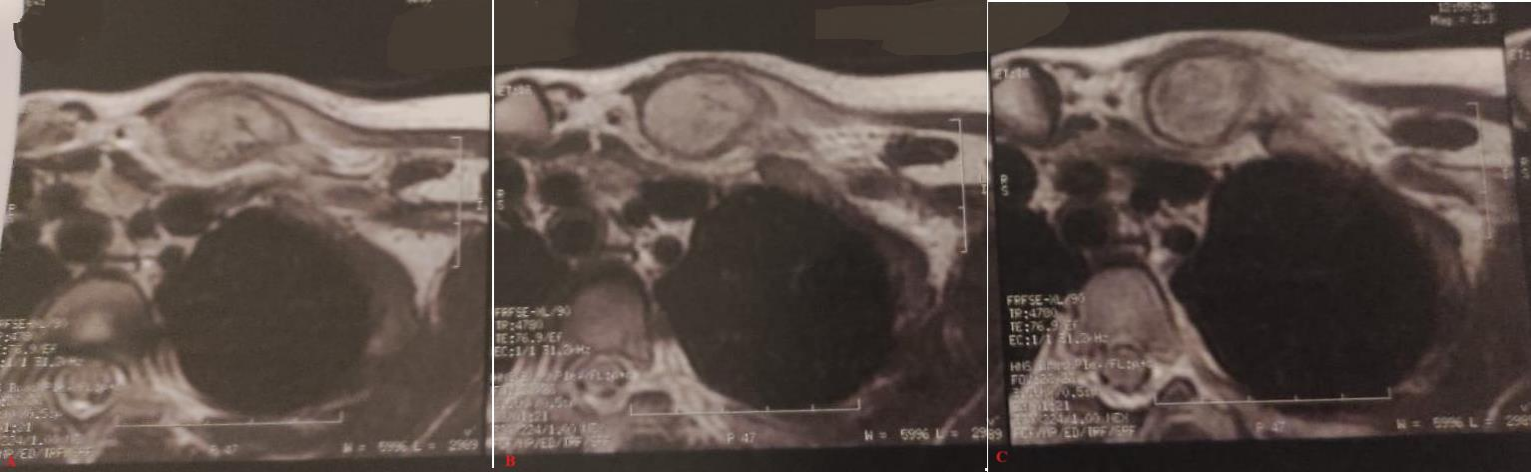
A,B,C) clavicle magnetic resonance imaging: axial section; low intense lesion in T1-weighted images and high intense in T2-weighted, heterogeneously enhancing, edematous signal of the surrounding soft

**Figure 4:**
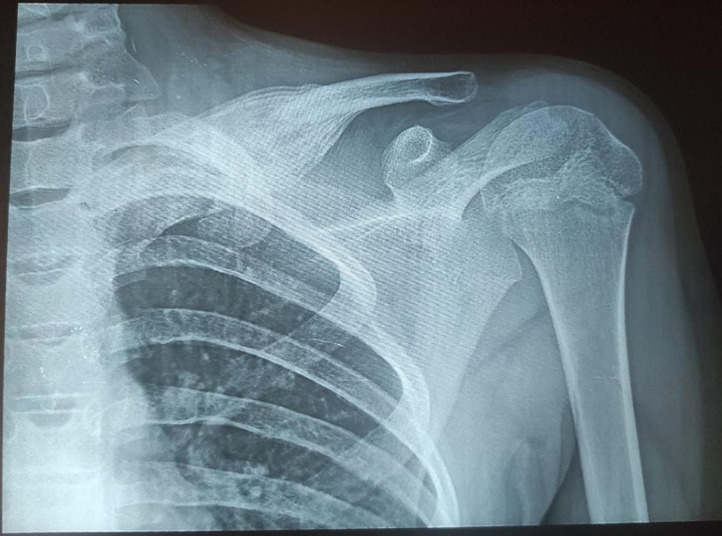
anteroposterior control shoulder X-ray: periosteal clavicle apposition with “onion bulb” shape lesion

**Diagnostic assessment:** an osteolytic lesion on the medial part of the left clavicle with cortical disruption was noted on a shoulder X-ray (Figure 2). inflammatory markers were negative (white blood cells count was 9250 cells per mm^3^, creatinine reactive protein (CRP) was 8 mg/L, and the erythrocyte sedimentation rate (ESR) was 12 mm first hour). Wright serology was negative. Ultrasonography revealed a sleeve around the medial end of the left clavicle with irregularities in the bone cortex. MRI showed an extensive lesion measuring 9 x 2 x 2 cm located at the medial end of the left clavicle and extending to the acromioclavicular joint (Figure 3). A needle biopsy was initially performed, suspected bone infection and the child received antibiotics with no recovery and appearance of important periosteal apposition on radiographs (Figure 4). Ewing's sarcoma was still suspected and a second open biopsy was performed two weeks later as well as the culture of mycobacteria and the polymerase chain reaction (PCR) for the Koch bacillus.

**Figure 5:**
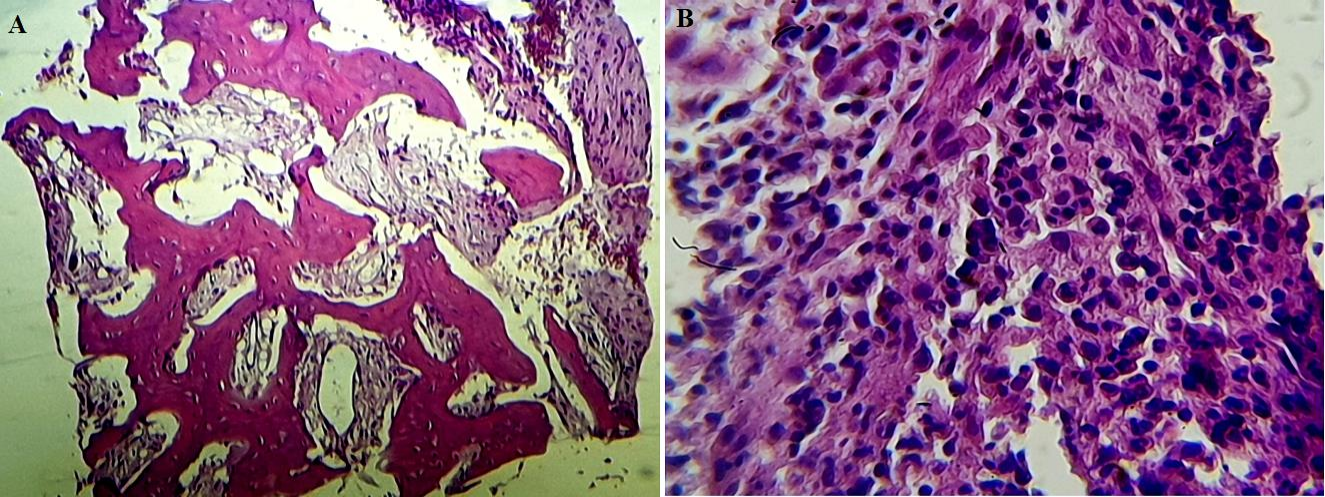
A) hematoxylin-Eosin staining x200: Trabecular architecture of bone; B) hematoxylin-Eosin staining x400: inflammatory cell infiltration mainly including neutrophils

**Figure 6:**
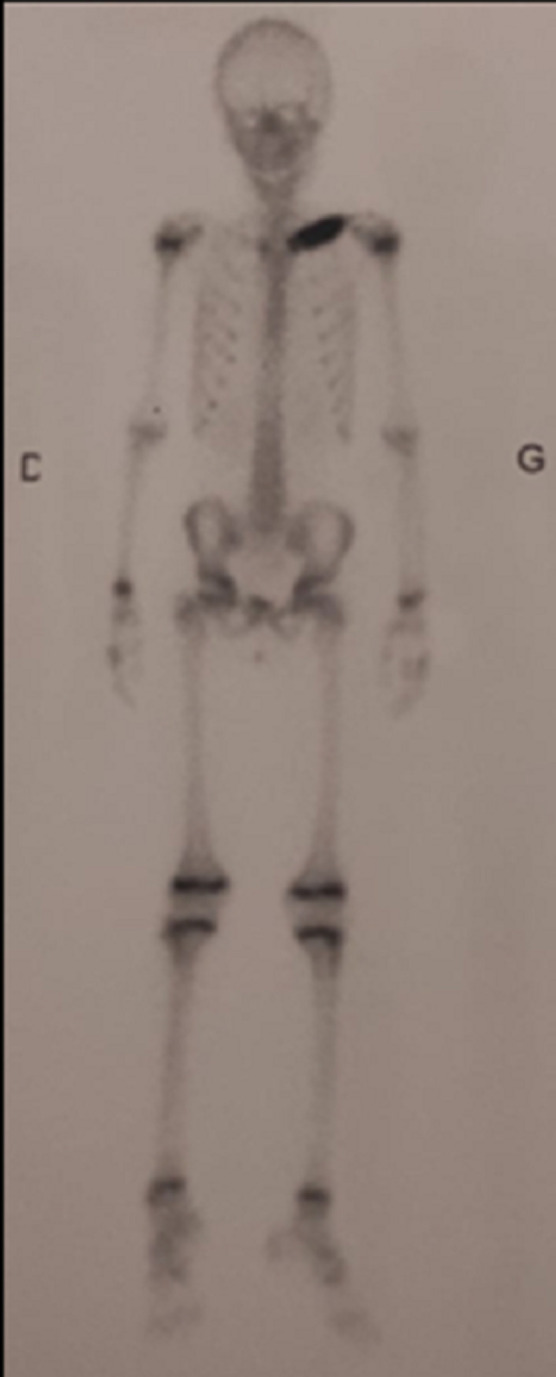
bone scintigraphy: isolated isotop uptaking on the inner part of the left clavicle with no other location

Histology study reveals no tumor cells but a fibroblastic component filling the intertrabecular spaces. An inflammatory neutrophils and osteoclasts cells infiltration was observed (Figure 5). CD1a was negative in the immunohistochemical study, which eliminated Langerhansian histiocytosis. Bone scintigraphy was performed and showed increased uptake on the inner part of the left clavicle with no other locations (Figure 6).

**Diagnosis:** chronic recurrent multifocal osteomyelitis diagnosis was sustained based on osteolytic bone lesion, normal blood count, good general health state and bone biopsy excluded sarcoma and showed no specific osteitis.

**Therapeutic interventions:** the child received a non-steroidal anti-inflammatory drug (NSAID): naproxen at a dose of 5 mg/Kg for three months.

**Follow-up and outcome of interventions:** spectacular improvement of pain after two weeks of NSAID and resumption of normal sports activities. The swelling of the clavicle disappeared after 10 weeks. The child remains asymptomatic and in excellent clinical condition, with no reported recurrence.

**Patient perspective:** “*I can now play basketball at school with my friends*”.

**Informed consent:** the patient’s parents gave informed consent for using the data file for scientific publication. Authors certified that their child couldn´t be recognized in the clinical photo.

## Discussion

Chronic recurrent multifocal osteomyelitis (CRMO) is a chronic non-microbial osteomyelitis. It's a rare disease with a prevalence of 1 to 2/10^6^ [4]. This disorder affects children and adolescents, more often females [4]. It belongs to the juvenile form of synovitis, acne, palmoplantar pustulosis, hyperostosis, and osteitis (SAHPO) group syndrome. Manifestations could be unique or multifocal. Typical locations are long bones metaphysis (74%), pelvis (38%), spine (46%), clavicle (25%), mandible (18%), sternum (8%), and ribs (8%) [2]. Isolated involvement of the clavicle presented in our case is typical of what has been described as Friedrich's disease [5]. Chronic recurrent multifocal osteomyelitis pathogenesis is still unclear and may be related to the imbalance between pro-inflammatory cytokines (IL-6, IL-1, TNF α) and anti-inflammatory cytokines (IL-10). These cytokines are involved in bone resumption and remodeling through osteoblasts and osteoclasts activation [6].

Clinical presentation often involves bone pain, swelling, inflammatory joint signs as in our case, and sometimes fever [1]. A mildly elevated ESR is the only abnormality that can be observed in patients with CRMO. Some patients may have higher white blood cell count or elevated CRP [7]. Radiologic signs are various and non-specific remains often normal in the early stage of the disease. In the later stage osteolytic and hyperostosis bone reaction could be noted as in our child after one month of spontaneous evolution. Scintigraphy shows in addition to bone isotope uptake in painful areas other sites in multifocal form. MRI remains sensitive but not specific for CRMO diagnosis (inflammatory bone signal) with enhancement after gadolinium injection. Whole-body MRI, non-radiating imaging, is more sensitive than bone scintigraphy in detecting clinically asymptomatic lesions [8]. Biopsy is still controversial because histological features are not specific (inflammatory infiltrates with neutrophils, lymphocytes, plasma cells, and histiocytes as well as osteolysis, and sclerosis [1]) but it helps to exclude infectious osteomyelitis, and malignant bone tumors, especially in a single bone lesion as in our patient. Mycobacterial detection is consistently negative [8]. Chronic recurrent multifocal osteomyelitis diagnosis is challenging and can be based on major and minor clinical imaging, and histopathology criteria established by Jansson *et al*. [9,10]. Diagnosis could be retained if there are two major criteria or one major and three minor criteria [10]. Our case associates two major and two minor criteria.

There are no consensus recommendations for CROM treatment. Non-steroidal anti-inflammatory drugs are given as the first intention in therapeutic management to control pain and prevent bone damage. Corticosteroids are used in patients who are resistant to NSAIDs. Methotrexate represents a second-line treatment. Sulfasalazine is generally used in patients with associated inflammatory bowel disease [7].

## Conclusion

Chronic recurrent multifocal osteomyelitis (CRMO) of the clavicle in a 9-year-old child with delayed diagnosis in this unusual location. Clinical and imaging signs were not specific miming bone tumors and infection with osteolytic bone lesion. Bone biopsy was helpful by excluding sarcoma. Non-steroidal anti-inflammatory drug treatment was effective and can be sufficient for recovery and remains the first recommended treatment.
